# Editorial: Surgical management of colorectal pathologies

**DOI:** 10.3389/fsurg.2025.1607005

**Published:** 2025-04-28

**Authors:** Peter C. Ambe, Michail Karanikas, Selman Sokmen

**Affiliations:** ^1^ Herdecke University, Witten, Germany; ^2^ Democritus University Thrace, Komotini, Greece; ^3^ Head of Colorectal & Pelvic Surgery Unit, Department of Surgery, Chairman of Peritoneal Surface Malignancy Center, Dokuz Eylul University Medical Faculty, Izmir, Türkiye

**Keywords:** colorectal cancer, laparoscopic oncologic surgery, appendicitis, diverticular disease, oncologic resection

**Editorial on the Research Topic**
Surgical management of colorectal pathologies

The surgical management of colorectal pathologies constitutes a huge aspect of the surgical workload in daily practice. Understanding the evolution in the trends of colorectal surgery is paramount in stay on top of our rapidly evolving specialty. This is only possible by constantly updating our knowledge on both benign and malignant conditions of the colon and rectum.

The recently completed research topic on the “*Surgical Management of Colorectal Pathologies*” highlighted recent developments in the management of both benign and malignant conditions of the anorectum. Twenty-one manuscripts looking at different aspects on colorectal pathologies were submitted and nine were accepted for publication following peer review. This acceptance quotient reflects the high standards set by the editors and esteemed reviewers while working on this topic. This editorial briefly summarizes the articles published in the research topic:

Laparoscopic appendectomy is probably one of the most commonly performed procedures in general surgery. Despite being a common procedure, the best means of approaching the appendix during laparoscopic appendectomy remains a matter of debate. The retrograde technique is performed be creating a small peritoneal window within the mesoappendix at the basis of the appendix prior to division of the appendix. This technique was compared with the anterograde technique by Ko et al., indicating that retrograde dissection was significantly longer than antegrade dissection (34.85 min vs. 40.92 min, *p* = 0.002). However, there was no statistically significant difference amongst both techniques with regards to perioperative complications. While the meaning of a delta of about six minutes between both techniques remains questionable, this study demonstrates the safety of the retrograde technique of laparoscopic appendectomy (Ko et al).

The surgical management of complicated diverticular disease can be very challenging. Amongst the complications of diverticular disease, colovesical fistula warrants special attention due to the involvement of both the bowel and the urinary system. Rizzuto et al. reported their experience shifting from an open to a laparoscopic approach, indication the advantages of minimally invasive access in this challenging surgical population (Rizzuto et al.).

In a study with the title “*Microsatellite Instability is highly prevalent in older patients with Colorectal Cancer*”, Jakob et al. questioned the practice of performing screening for microsatellite instability (MSI) in an age – based manner. The authors found MSI-H tumor in 18.2% of cases >50 years, and in 20.6% of patients >60 years in their collective. The authors argued that both the role of MSI-H as an indicator of a hereditary cancer predisposition (Lynch Syndrome) as well as its relevance in the decision-making with regard to the need and choice of additive chemotherapy should warrant a systematic screening, independent of age and clinical criteria (Jakob et al.).

Malignant colonic obstruction remains a serious complication of colorectal cancer and it´s management can not only be challenging but may also be associated with poor overall outcome. Evidence-guided management is literally not available, and the current clinical practice is mostly guided by small retrospective series with well known flaws. Compiling existing data to help guide clinical decision-making in the critical subset of patients. This clinical meaningful task was undertaken by Mikalonis et al. in the article titled “*Danish guidelines for treating acute obstruction caused by colorectal cancer – a review*” (Mikalonis et al.).

Besides bowel obstruction, distance metastasis is not uncommon in patients with CRC. About 20% of patients with CRC present with hepatic lesions at the time of diagnosis and about 50% is expected to develop liver metastasis in the course of time. Of clinical importance is also the observation of recurrence following partial hepatectomy. This important cancer dynamic was investigated in a mouse model by Luenstedt et al., indicating that regenerative pathways secondary to partial hepatectomy may lead to accelerated colorectal metastasis by priming a premetastatic niche in the liver (Luenstedt et al.).

Focusing on the right colon, Qin et al. used the SEER database to design and test a prognostic nomogram for survival in patients with right-sided colon cancer after colectomy. The authors identified age, chemotherapy, CEA and disease stage per TNM classification as prognostic factors to develop a nomogram with high performance in prediction 1-year, 3-year and 5-year overall survival in patients following right-sided colectomy for colon cancer. The results reported in this study may be helpful in counselling patients e.g., with regards to the need of adjuvant chemotherapy and for follow – up after right colectomy for cancer (Qin et al.).

Total mesorectal excision (TME) represents the standard technique for radical resection of rectal cancer and the laparoscopic approach has been established a standard procedure. Laparoscopic TME requires a high level of expertise and thus may pose some degree of challenge. Thus, predicting the difficulty of surgery may ease decision-making with regard to patient selection. In the paper titled “*Interpretable machine learning model to predict surgical difficulty in laparoscopic resection for rectal cancer*” Yu et al. demonstrated an XGBoost model for predicting the difficulty of laparoscopic TME, thus providing a useful tool to help surgeons select appropriate candidates for laparoscopic TME (Yu et al.).

Postoperative pain management represents an important aspect of enhanced recovery after surgery (ERAS). However, striking a balance between pain management and medication -induced adverse events, including bowel paralysis following colorectal surgery, may be challenging. In the RCT by Cao et al., the efficacy of postoperative pain control using a combination of ropivacaine and parecoxib was compared with patient controlled intravenous analgesia (PCIA) consisting of 100 ug sufentanil and 16 mg ondansetron after laparoscopic surgery for CRC. The study endpoint included pain measured via the VAS as well as biochemistry markers including Interleukin 6 (IL-6) and C-reactive protein (CRP). The results of this RCT confirmed a statistically significant reduction in postoperative pain control using PCIA. This trend correlated with a significantly lower expression of IL-6 in the PCIA group. As expected, there was no statistically significant difference amongst both groups with regard to postoperative CRP. This RCT addresses two important issues: First, PCIA is associated with effective postoperative pain control and should be part of standard ERAS programs following colorectal resection and second, IL-6 may represent an objective tool for measuring postoperative pain (Cao et al.).

In the manuscript with the title “*Effect of prehabilitation exercises on postoperative frailty in patients undergoing laparoscopic colorectal cancer surgery*” Yang et al. explored a new intensive prehabilitation program that combines prehabilitation exercises with stand enhanced recovery after surgery on frailty in a randomized controlled trial (Yang et al.). The study indicated that prehabilitation exercises can improve postoperative frailty and accelerate recovery in elderly patients undergoing laparoscopic oncologic colorectal resections. This is a meaningful finding in light of the changing global demographics with an increasingly aging population.

While only 43% of all submissions was accepted for publication, the editors and reviewers applauded all submitting groups for their contribution to the success of this special issue. More importantly, all manuscript that didńt qualify for publication received fair-minded comments to help the authors improve their work.

